# Unraveling Immune Dysregulation in ZNFX1 Deficiency: From Interferonopathies to Macrophage Activation Syndrome

**DOI:** 10.1007/s10875-026-02056-4

**Published:** 2026-08-06

**Authors:** Sara G. Hamad, Melanie Makhlouf, Nourhen Agrebi, Ahmad Kaddourah, Mutasim Abu-Hasan, Buthaina Al-Adba, Ameera Sadoun, Satanay Z. Hubrack, Waleed Aamer, Mayra L. Ruiz Tejada Segura, Antonio Scialdone, Khaled Machaca, Luis R. Saraiva, Sharon Bout-Tabaku, Bernice Lo

**Affiliations:** 1 Department of Pediatrics, Hamad Medical Corporation, Doha, Qatar; 2 Research Branch, Sidra Medicine, Doha, Qatar; 3 Department of Pediatrics, Sidra Medicine, Doha, Qatar; 4 Department of Pediatrics, Weill Cornell Medicine Qatar, Doha, Qatar; 5 University of Qatar, Doha, Qatar; 6 Institute of Epigenetics and Stem Cells, Helmholtz Zentrum München, Munich, Germany; 7 Institute of Functional Epigenetics, Helmholtz Zentrum München, Neuherberg, Germany; 8 Institute of Computational Biology, Helmholtz Zentrum München, Neuherberg, Germany; 9 Calcium Signaling Group, Research Department, Weill Cornell Medicine-Qatar, Doha, Qatar; 10 Department of Physiology and Biophysics, Weill Cornell Medicine, New York, NY USA; 11 College of Health and Life Sciences, Hamad Bin Khalifa University, Doha, Qatar; 12 Department of Comparative Medicine, Yale University School of Medicine, New Haven, CT USA

**Keywords:** Interferonopathies, Macrophage activation syndrome, Immune dysregulation, Autoinflammatory disease, Thrombotic microangiopathy, Interferon stimulated genes (ISGs)

## Abstract

**Purpose:**

ZNFX1 interferes with early viral replication after exposure to type I interferon, and interacts with mitochondrial antiviral sensors (MAVS) leading to a balanced expression of interferon-stimulated genes (ISGs). ZNFX1 deficiency mimics systemic autoinflammatory diseases and accurate identification may prevent early mortality. We compare a homozygous *ZNFX1* frameshift mutation patient to subjects with known interferonopathies. We explore the association between this mutation, unregulated ISGs, and macrophage activation syndrome (MAS).

**Methods:**

Study participants included our index patient, other patients with well-characterized interferonopathies, and healthy participants. We performed western blotting for ZNFX1 and whole Blood mRNA-seq, RT-qPCR, ELISA, and Olink Proteomics analysis to assess cytokine production and the underlying immune dysregulation.

**Results:**

Our patient had elevated expression of interferon-inducible genes compared to healthy controls, but levels were comparable to subjects with known interferonopathies. Gene sequencing revealed a novel homozygous *ZNFX1* frameshift variant resulting in a truncated protein. Transcriptomic analysis pointed to the top 3 cytokine upstream regulators, tumor necrosis factor (TNF), IL-1β, and interferon-gamma. ZNFX1 deficient patients appear to have a clinical profile that differs from classical type I interferonopathies, including features such as thrombotic microangiopathy (TMA) and a higher frequency of MAS.

**Conclusions:**

ZNFX1 deficiency is an immune dysregulation disorder characterized by a robust type I interferon signature combined with elevated inflammatory cytokines, leading to severe early-onset multisystem inflammatory disease. Characterizing the immune profile allows targeted short-term treatments that may prolong life.

**Supplementary Information:**

The online version contains supplementary material available at https://doi.org/10.1007/s10875-026-02056-4.

## Introduction

The discovery of monogenic immune dysregulation and autoinflammatory diseases have increased in the past years. They have been characterized by gain-of-function and loss-of-function mutations in genes that affect different immune response pathways. One pathway is through interferon (IFN) signaling which leads to dysregulated, chronic IFN signaling. Successful therapeutic interventions that interfere with interferon signaling through the Janus kinase-signal transducer and activator of transcription (JAK-STAT) pathway provided evidence of the mechanism of interferon-mediated diseases. Interferonopathies such as Sting-associated vasculopathy with onset in infancy (SAVI), Proteasome- Associated Autoinflammatory Syndromes (PRAAS), and Aicardi Goutières syndrome (AGS) are examples of chronic, abnormal interferon signaling due to different mechanisms, with similar pathogenic effects of unregulated Type I interferon (IFN) such as excess inflammation and tissue destruction [1].

Mutations in *ZNFX1* have been reported to cause a severe multi-system inflammatory disease [2–7]. When functioning normally, ZNFX1 interferes with early viral replication after exposure to type I interferon, and interacts with mitochondrial antiviral sensors (MAVS) leading to a balanced expression of interferon-stimulated genes (ISGs) [8]. Mutations in *ZNFX1*, interfere with the viral recognition response, resulting in poor viral clearance, and altered post-translational regulation of ISGs [7]. The half-life of ISG expression is extended upon stimulation with intracellular DNA, leading to a stronger inflammatory response than seen with a viral clearance response [2, 7]. We present a case of a child with a homozygous *ZNFX1* frameshift mutation, with systemic inflammation causing multiorgan disease, and frequent, severe viral infections. We compared transcriptomic and inflammatory profiles from a patient with ZNFX1 deficiency to those from patients with interferonopathies and healthy controls. Furthermore, we explore the association between this mutation, and unregulated ISGs, and macrophage activation syndrome (MAS), a form of secondary hemophagocytic lymphohistiocytosis (sHLH).

## Methods

All study participants and their legal guardians provided written informed consent. Blood samples and clinical data were collected under an Institutional Review Board approved protocol (Sidra Medicine IRB, Protocol 1500768, approved June 30, 2016).

## Whole Genome Sequencing

Genomic DNA from the family were whole genome sequenced in the Sidra Medicine Integrated Genomic Services core facility to an average depth of 30 reads per base using the Illumina sequencing platform. Reads were aligned to the human genome reference version *hg19* using the Burrows-Wheeler Aligner. Variants were called using the Genome Analysis Toolkit (GATK) HaplotypeCaller and annotated using SnpEff/SnpSift. Variants were prioritized based on quality scores, mode of inheritance (i.e., recessive or de novo), allele frequency below 0.005 in gnomAD, in silico deleteriousness predictions (e.g. CADD, PolyPhen, SIFT, MutationTaster), and relevance to the clinical phenotype. The *ZNFX1* c.3408dupT variant was confirmed by Sanger sequencing.

## Whole Blood mRNA-seq

Whole blood from patients and healthy donors were collected in PAXgene RNA tubes (Qiagen). RNA was extracted using the PreAnalytiX PAXgene Blood RNA kit according to manufacturer’s instructions. RNA-seq libraries were prepared using Illumina TruSeq mRNA library kit and sequenced to an average depth of 50 million reads per sample using the NovaSeq platform (Illumina). STAR_2.6.1d aligner was used to map reads to GRCh38.p13 and raw counts were generated using FeatureCounts v.2.0.0.

Samples were sequenced on three Illumina platforms (NextSeq500, HiSeq4000, and HiSeqX) across multiple runs between 2019–2021. Sequencing platform was modeled as a batch covariate in the DESeq2 design (~ batch + condition), and the effectiveness of batch correction was validated by variance-stabilizing transformation and principal component analysis (PCA). Sample-level metadata, including sequencing platform, run date, is provided in Supplementary Table S1.

## Differential Expression Analysis and Functional Annotation

Transcriptome data quality was assessed by evaluating homogeneity and similarity between samples. We used variance stabilizing transformation(vst) and regularized log transformation(rlog) functions (DESeq2 package (v1.26.0)) to transform the raw count data, (dist) function to calculate sample-to-sample distances. We plotted heatmaps of the distance matrix and principal component analysis (PCA) of 1000 most highly-variable genes to identify outliers. We used DESeq2 (v1.26.0) to normalize the raw counts according to sequencing depth and RNA composition (median of ratios method), and to perform differential expression analysis comparing the ZNFX1 patient samples to other patient samples and/or healthy control samples.

We ensured that the same sequencing platforms were represented in both groups of samples being compared. Genes not expressed in any of the taken samples were removed from the dataset (Fig. S1A, Fig. S2A in the Online Resource). Differential expression was tested using the DESeq function from the DESeq2 library. Finally, DESeq was used in FDR correction for multiple testing [9]. Genes with an FDR < 0.05 and |log2FC|> 1 were considered as differentially expressed.

## Functional Annotation of DEGs

Gene over-representation analysis was performed using ClusterProfiler package (v4.0.0) [10]. We used Gene Ontology enrichment (enrichGO) and Simplify functions, with ont = "BP" and pAdjustMethod = "BH" parameters to assess which Biological processes were affected in our lists of differentially expressed genes. We performed pathway enrichment analysis and Upstream regulator analysis using expression core analysis on expression log ratios using the Qiagen Ingenuity Pathway Analysis software (QIAGEN Inc., https://digitalinsights.qiagen.com/IPA). Pathways and Upstream Regulators with Benjamini–Hochberg p-value adj < 0.05 were considered statistically significant. Summary statistics for the differential expression analyses are provided in Supplementary Table S2. The quantitative metrics for the pathway and upstream regulator analyses are provided in Supplementary Table S3.

## ELISA and Olink

Plasma was collected from patient and healthy donors in sodium heparin tubes. Plasma IL-1β levels were measured using the Human IL-1β ELISA (HRP) (3416-1H, Mabtech AB, USA) according to the manufacturer's instructions. Plasma cytokines and chemokines were measured using the Olink Target 96 Inflammation panel (Sweden) according to manufacturer’s instructions and included the use of internal and external quality controls. The reporter DNA levels bound to the cytokine-specific antibodies were measured on the 96-well Fluidigm integrated chip (San Francisco, CA, USA), and the Fluidigm RT-PCR analysis software was used to determine RT-PCR cycle threshold (Ct) values using a quality threshold of 0.5 and linear baseline correction. Ct values were normalized to internal and external controls using the Olink NPX Manager software and transformed into log2-relative protein levels.

## Generation of Epstein − Barr virus-immortalized B lymphocytes (EBV + B cells)

Peripheral Blood Mononuclear Cells (PBMCs) (1–2 million) in 1 ml of RPMI 1640 supplemented with 20% FBS, cyclosporin A were infected with EBV using 1 ml of thawed B95-8 culture supernatant. Fresh culture media was supplemented periodically starting a week post-infection and until stable cell proliferation is established (generally a month).

Additional methodological details are provided in the Supplementary Section Methods in the Online Resource.

## Results

### Clinical Presentation

A two-month-old, full-term female, presented to our pediatric hospital with bloody diarrhea, petechial skin rash, hypoactivity, and poor feeding. The family history was significant for consanguinity and stillbirth.

**Fig. 1:**
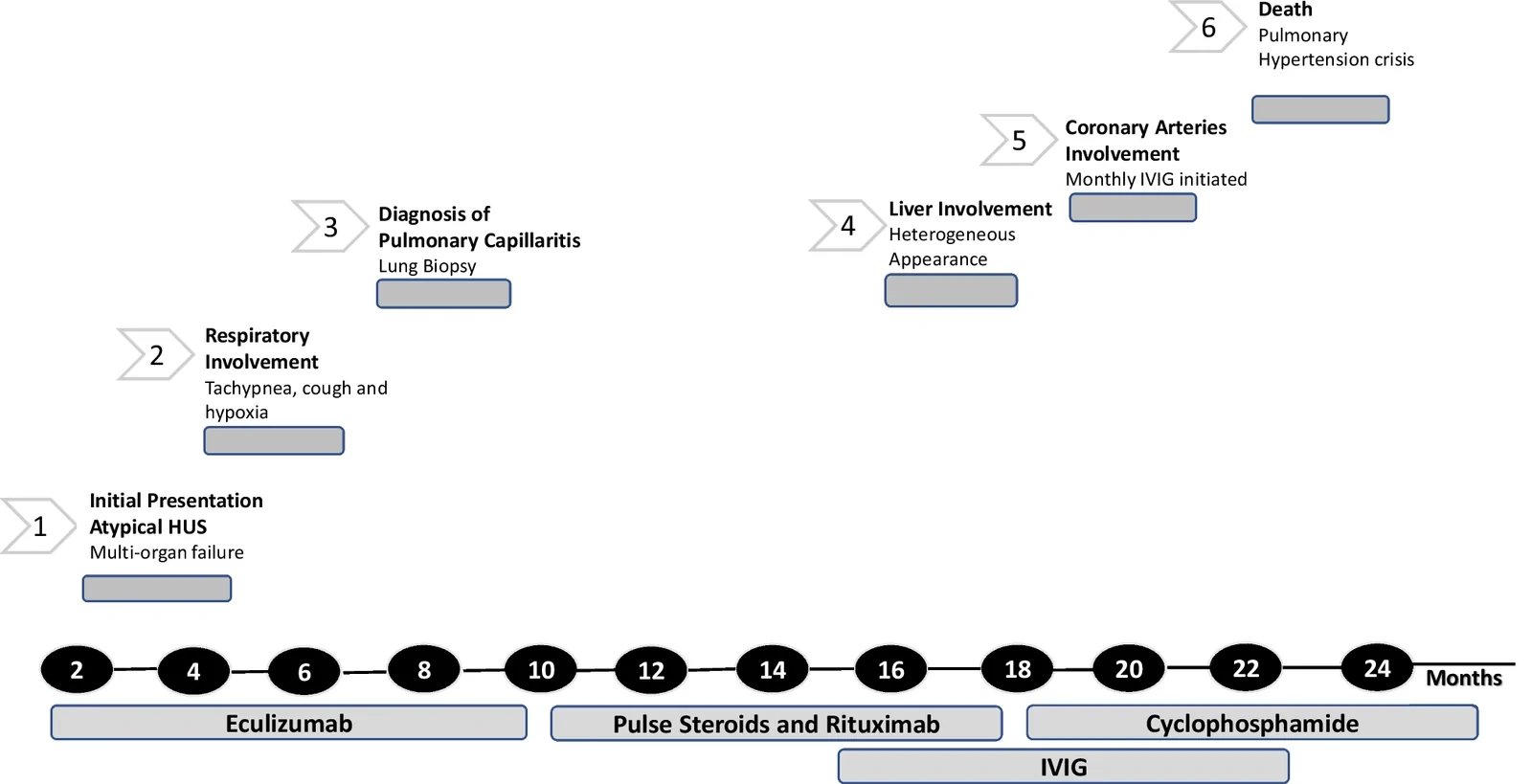
Clinical course of the patient with significant multiorgan disease

She presented in hypotensive shock and had hepatosplenomegaly. Her white blood cell count was 48.8 × 10^9^/L with a 50% lymphocytic predominance, hemoglobin of 8.2 g/L, severe thrombocytopenia (13 X 10^9^/L), and metabolic acidosis with pH of 7.04, and serum bicarbonate of 12 mEq/L. Her CRP was 59.9 mg/L, and HSV-1 and 2 and Enterovirus blood PCR were positive. The remaining blood, fungal and urine cultures were all negative. She was diagnosed with septic shock and broad-spectrum antibiotics were started. She developed stage 3 acute kidney injury, severe transaminitis, hyperferritinemia, coagulopathy, hypoalbuminemia, and hyperammonemia. Hemolysis was evident with undetectable haptoglobin levels, LDH > 2000 IU/L, and schistocytes in the peripheral smear, suggesting thrombotic microangiopathy (TMA) (Table 1). Empiric intravenous corticosteroids, intravenous immunoglobulin (IVIG), and a metabolic cocktail were started, followed by plasma exchange and continuous kidney replacement therapy (Fig. 1).

**Table 1:** ZNFX1 mutation compared to patients with MAS and HLH

	MAS in sJIA (Ravelli 2016)	HLH criteria (2004)	Our patient
Fever	+	+	+
Hepatomegaly	-	-	+
Splenomegaly	-	+	+
Bicytopenias	-	+	+
WBC	-	-	48.8 × 10^9/liter
Neutrophils	-	< 1 × 10^9/liter	12.5 × 10^9/liter
Hemoglobin	-	< 90 g/liter	82 g/liter
Platelets	≤ 181 × 10^9/liter	≤ 100 × 10^9/liter	13 × 10^9/liter
Ferritin	> 684 µg/liter	≥ 500 µg/liter	4351 µg/liter
Hypertriglyceridemia	> 1.8 mmol/liter	≥ 3.0 mmol/liter	1.4 mmol/liter
Hypofibrinogenemia	≤ 3.6 g/liter	≤ 1.5 g/liter	0.7 g/liter
Liver dysfunction	AST > 48 U/liter	-	AST 197 U/liter
Soluble CD25	-	≥ 2400 U/ml	Not done
Hemophagocytosis	-	+	-
	Known or suspected JIA, fever and increased ferritin combined with > 2 lab criteria	Diagnosis requires molecular diagnosis or > 5 of 8 diagnostic criteria. Malignancy excluded

By Day 9, she improved significantly with lower LDH levels, resolving thrombocytopenia, and recovering kidney function. Mutations for metabolic and primary hemophagocytic lymphohistiocytosis (pHLH) were negative. Complement pathway function workup showed low factor H, elevated terminal C5-9 complement complex, and depressed alternative complement pathway (AH50), suggestive of alternative complement pathway dysregulation.

Next-generation targeted sequencing for an Atypical Hemolytic Uremic Syndrome (aHUS) panel showed no coverage for the complement factor H related protein 1 and 3 (CFHR1 and CFHR3) genes. Homozygous deletions in CFHR3 and CFHR1 genes were identified, suggestive of alternative complement pathway activation. The deletion was confirmed on chromosome 1 by PCR according to previously described methodology [11]. Clinical whole exome sequencing (WES) showed seven variants of uncertain significance unrelated to the disease. Thus, she was diagnosed with aHUS and started on eculizumab, an anti-complement C5 antibody. She improved, and was discharged home on eculizumab.

**Fig. 2:**
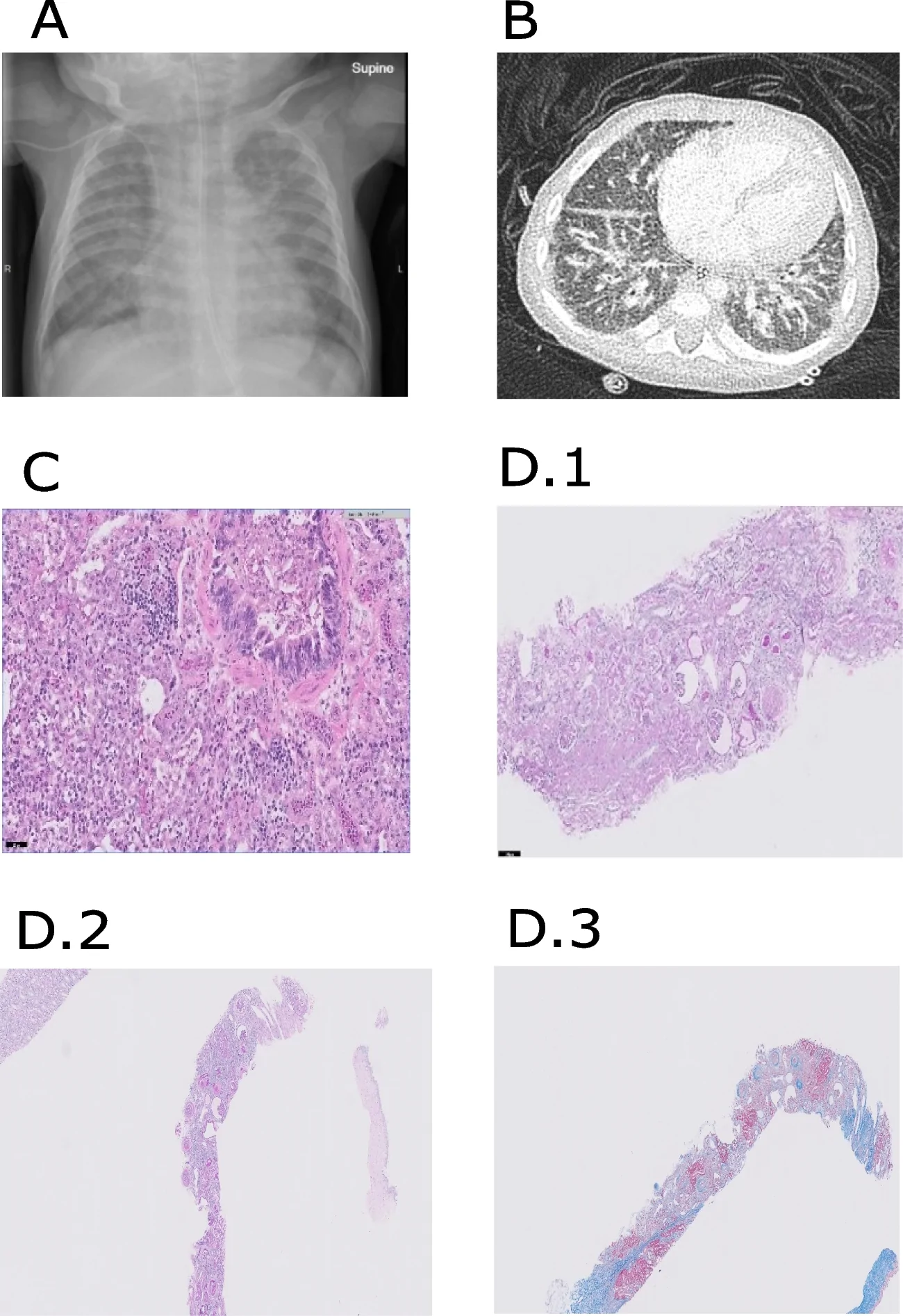
Radiologic and histopathologic findings demonstrating pulmonary and renal involvement. **A** Chest Xray at 6 months of age: Diffuse patchy interstitial and airspace opacities. **B** CT Chest at 6 months of age: Extensive lung parenchymal changes with patchy ground glass changes and some nodularity; interstitial changes with interlobular septal thickening. **C** Lung Biopsy: Peribronchial inflammation with capillaritis, some pulmonary hemosiderosis and focal bronchiolitis. No alveolar proteinosis or organisms were identified. Stains used were Hematoxylin and Eosin (H&E). **D** Kidney Biopsy: Glomerular sclerosis, mild mesangial increase with hyaline thick capillary loops and mesangial matrix. Interstitium shows patchy fibrosis with minimal inflammation and tubular atrophy. The PAS silver stains show some focal thickening of the basement membrane. Stains used were (1) H &E, (2) PAS silver stain and (3) Masson’s trichome

At six months of age, she presented with two months of dry cough and tachypnea. Her oxygen saturation was 86% on room air, she had diffuse crackles and her hemoglobin was 7.4 g/dL. There was no evidence of aHUS activation, her kidney function was normal, and there was no evidence of complement activation. The eculizumab levels were therapeutic. Chest x-ray showed diffuse interstitial and airspace opacities, and CT showed diffuse extensive ground-glass opacities (Fig. 2A-B). Broncho-alveolar lavage (BAL) had abundant hemosiderin-laden macrophages, and the viral respiratory PCR panel, bacterial, and fungal cultures were all negative. Antinuclear antibody (ANA), anti-glomerular basement membrane (Anti-GBM), and antiphospholipid antibodies were negative, but antineutrophil cytoplasmic antibody (ANCA) and double-stranded DNA (dsDNA) antibodies were weakly positive. Lung biopsy showed extensive peri-bronchial capillaritis with neutrophilic predominance, and no organizing pneumonia (Fig. 2C). Kidney biopsy showed glomerular sclerosis with no vasculitis or thrombotic microangiopathy (Fig. 2D), and immunohistochemistry was negative. Electron microscopy showed a normal basement membrane with no electron-dense deposits, a focal loss of foot processes with effacement, and fibrosis with mesangial extension and primitive appearing podocytes.

Intravenous pulse methylprednisolone was administered over three days for pulmonary capillaritis, and she improved. Eculizumab was discontinued for a concern of drug-induced capillaritis, and because she no longer had hemolysis or TMA, and kidney function was normal. Lymphocyte subsets (LSS) at 3 months showed 10% of B cells and 10% of NK cells. LSS at 6 months showed 53% of B cells and 5% of NK cells, and at 9 months, 63% B cells and 1.3% NK cells, respectively.

She was discharged on supplemental oxygen and oral prednisolone. Rituximab was given as a steroid-sparing agent, and to treat ANCA positive capillaritis. Her respiratory status improved, but the radiological abnormalities persisted. Over the next several months, she was hospitalized often with increased tachypnea and oxygen requirements due to respiratory viral infections. She received cyclophosphamide pulses, and remained on tapering doses of corticosteroids, but was never able to discontinue steroids. She never improved, had recurrent respiratory illnesses, including cytomegalovirus (CMV) viremia with a maximum viral load of 10,000 IU/mL, and cyclophosphamide was discontinued after 3 doses.

At nineteen months of age, she developed chronic diarrhea and hepatomegaly. Magnetic resonance enterography showed a chronic heterogenous cirrhotic liver which was confirmed by serial ultrasounds.

At twenty months of age, she had fever, tachycardia, lip swelling, and mouth ulcers positive for herpes simplex virus (HSV). Echocardiography showed mildly dilated right coronary artery with Z-score + 2.8. She was treated for incomplete Kawasaki disease with IVIG and aspirin. Monthly IVIG was started as prophylaxis after the CMV and HSV infections.

At twenty-four months of age, she had a lower respiratory tract infection requiring respiratory support, intubation and inhaled nitric oxide. She developed bradycardia, asystole, and failed cardiopulmonary resuscitation.

## Elevated Expression of Interferon-Inducible Genes in Whole Blood

**Fig. 3:**
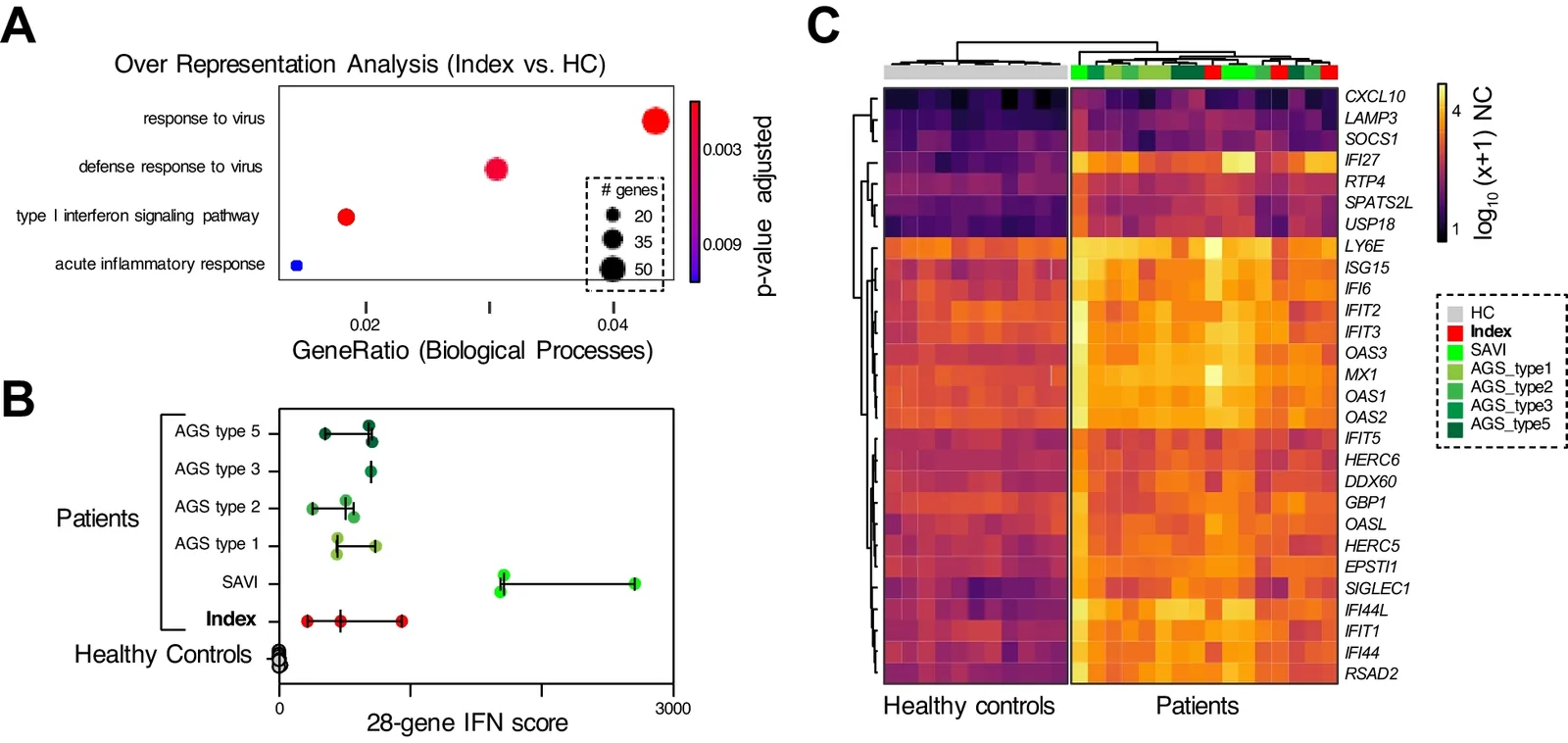
Whole blood transcriptomics analysis indicated an upregulation of ISGs in the index case similar to other interferonopathy patients. **A** Dot plot of four selected significant Gene Ontology terms from over-representation analysis of differentially expressed genes (index patient vs. healthy controls). Gene ratio represents the proportion of significant genes associated with each term; dot size indicates the number of enriched genes and dot color reflects the adjusted p-value. The enriched terms highlight a dominant antiviral and inflammatory transcriptional program in the index patient. **B** ISG score calculated from a 28-gene interferon signature in the index case, other interferonopathy patients (Aicardi-Goutières syndrome and SAVI), and healthy controls. Each point represents an individual sample; horizontal bars denote the mean ± SEM. Healthy controls show uniformly low ISG scores, while the index patient falls within the interferonopathy range, consistent with an interferon-high transcriptional state. **C** Unsupervised clustered heatmap displaying normalized ISG expression in the index patient, 13 interferonopathy patient samples, and 11 healthy controls. Expression values are shown as log10(normalized counts + 1). Samples and genes were clustered using Euclidean distance and Ward’s method. Highly expressed genes are shown in orange/yellow, whereas low or absent expression is shown in violet/black. The heatmap highlights the clear separation of healthy controls from patients and shows that the index patient clusters within the interferonopathy group

Due to the severe clinical phenotype occurring early in life, and multisystem organ disease, an autoinflammatory disorder was suspected. We performed whole blood mRNA-seq to characterize the nature of the inflammatory disease at 11, 14 and 15 months of age. Compared to healthy donors, she had significantly elevated expression of genes involved in host response to virus and type I IFN (Fig. 3A and 3B; Fig. S1A-E in the Online Resource) [12]. This interferon signature suggested overlap with type I interferonopathy-associated immune dysregulation. We compared the expression of ISGs in this index case with that of other patients with known interferonopathies, including SAVI and AGS (Fig. 3B-C). The levels of ISGs were lower than patients with SAVI, and comparable to patients with AGS. Hierarchical clustering analysis showed that her ISG expression pattern was similar to that of other interferonopathy patients compared to controls (Fig. 3C).

## Homozygous *ZNFX1* Frameshift Variant in Patient Results in a Truncated protein

**Fig. 4:**
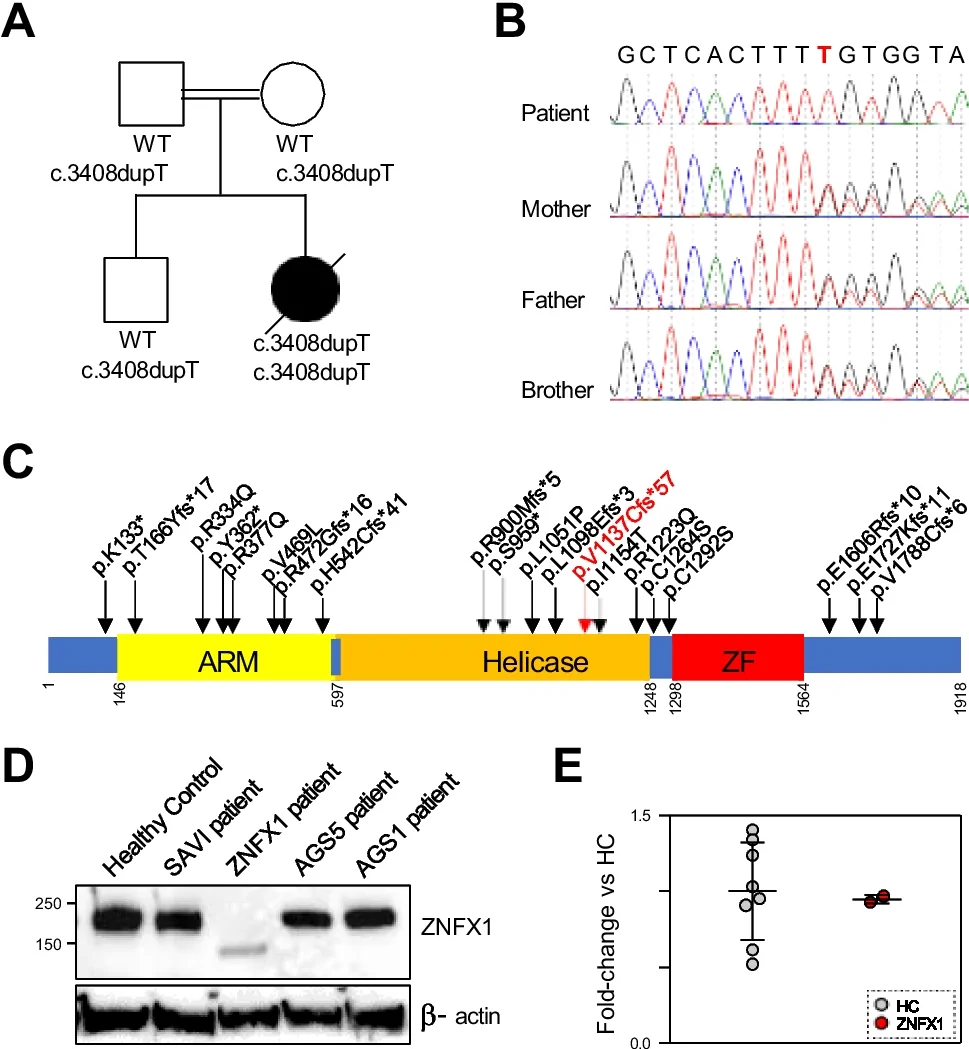
The *ZNFX1* homozygous frameshift mutation resulted in truncated protein. **A** Pedigree of a consanguineous family with 1 affected child homozygous for the *ZNFX1* c.3408dupT insertion variant, whereas both parents and healthy sibling are heterozygous for the *ZNFX1* variant. **B** Sanger sequencing analysis confirming the insertion variant in the patient compared to the heterozygous family members. **C** Schematic representation of ZNFX1 showing the armadillo (ARM), helicase, and zinc-finger (ZF) domains. Variant identified in this report (in red) and variants from previous studies (in black) are indicated. **D** Western blot of lysates from EBV + B cells from the ZNFX1 deficient patient compared to healthy donor and other interferonopathy patients. **E** ZNFX1 mRNA expression levels from EBV + B lymphocytes shown as fold-change of ZNFX1 mRNA-seq normalized counts from patient compared to healthy controls (HC)

We hypothesized that she had an interferonopathy, thus the patient, her parents and healthy sibling were whole-genome sequenced and a homozygous 1 base pair insertion in *ZNFX1* (c.3408dupT; p.Val1137CysfsTer57) was identified in the patient. Parents and the healthy sibling were heterozygous for the *ZNFX1* variant, which was confirmed by Sanger sequencing (Fig. 4A-B). The variant was not present in the Genome Aggregation Database (gnomAD) or the 1000 Genomes Project. The insertion variant was predicted to be a loss-of-function mutation that results in a frameshift and early stop codon in the P-loop helicase domain of ZNFX1 (Fig. 4C). To assess the impact of the variant on ZNFX1 protein expression, we performed Western blotting of cell extracts from Epstein − Barr virus-immortalized B lymphocytes (EBV + B cells) from the patient compared to healthy donors and patients with a known monogenic interferonopathy. The cell lysates from the patient with the *ZNFX1* mutation showed a truncated protein (Fig. 4D). RNA expression of the mutant *ZNFX1* was normal, indicating no nonsense-mediated decay (Fig. 4E).

## Elevated Inflammatory Cytokines and Pathway Activation in the ZNFX1-Deficient Patient

**Fig. 5:**
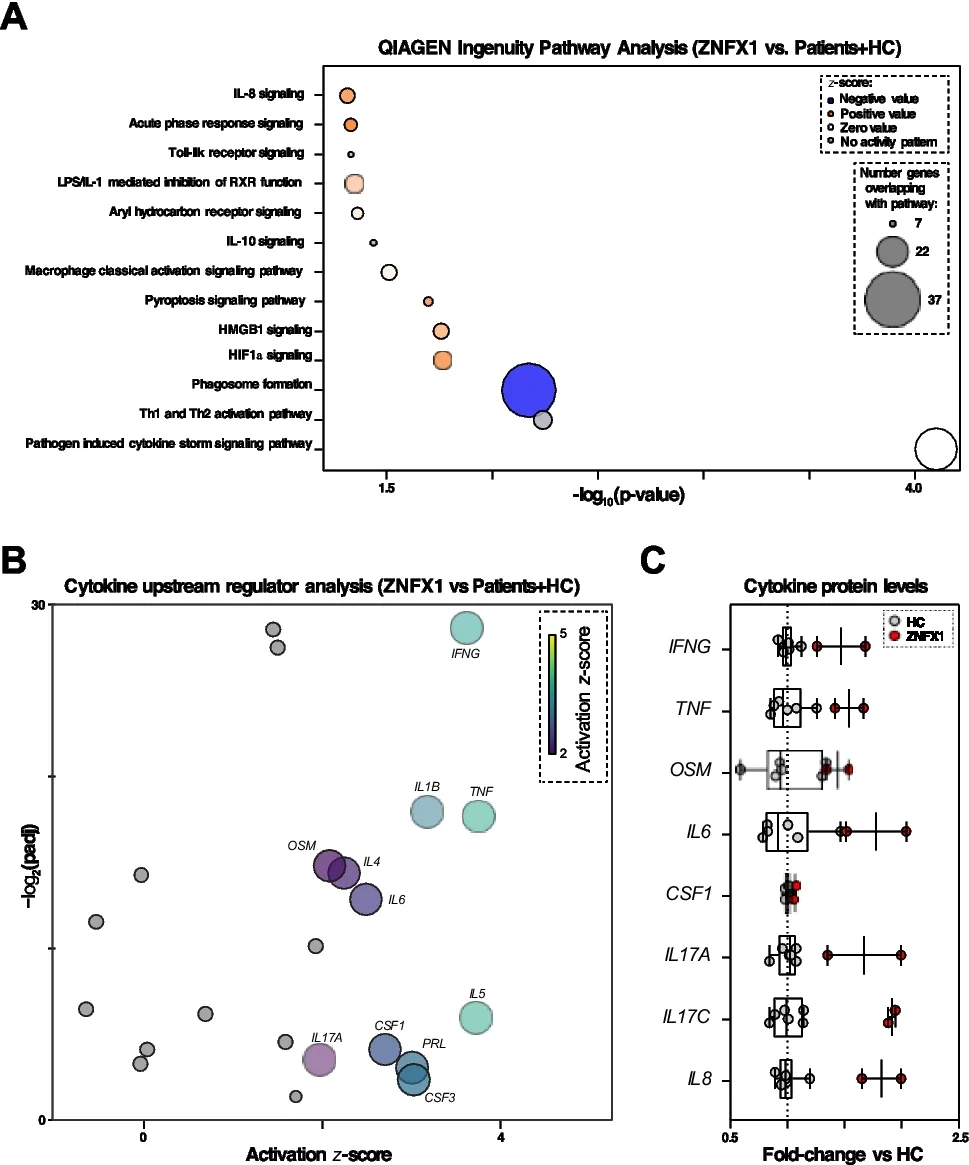
Inflammatory pathways upregulated in the ZNFX1-deficient patient compared to other interferonopathy patients and controls. **A** Canonical pathway enrichment in ZNFX1 deficiency (ZNFX1 vs. Patients + Healthy Controls). Dot plot of immune-related canonical pathways enriched among differentially expressed genes in the ZNFX1-deficient patient compared to interferonopathy patients and healthy controls. Pathways are ranked by -log_10_(p-value). Dot color reflects the IPA activation z-score (orange, predicted activation; blue, predicted inhibition; white, no predicted direction; gray, z-score not computed). Dot size indicates the number of ZNFX1-dysregulated genes overlapping each pathway. This analysis highlights a dominant myeloid-inflammatory program in ZNFX1 deficiency, including pyroptosis, macrophage activation, HMGB1, TLR, IL-8 and acute phase pathways**. B** Cytokine upstream regulator analysis (ZNFX1 vs Patients + Healthy Controls). Dot plot showing significant cytokine upstream regulators predicted by IPA to influence the transcriptional changes in the ZNFX1-deficient patient. The x-axis displays the activation z-score, and the y-axis shows the –log₂(adjusted p-value). Dot size reflects statistical significance, while dot color indicates the magnitude of predicted activation. Cytokines such as IFN-γ, TNF, IL-1β, IL-6, IL-4, and GM-CSF emerge as strongly activated regulators, highlighting a prominent pro-inflammatory cytokine network in the ZNFX1 deficient patient. **C** Bar plot reporting the plasma cytokine levels in ZNFX1 patient relative to the healthy controls using the Olink inflammation panel

To identify transcriptomic signatures unique to the ZNFX1-deficient patient, we determined the genes differentially expressed in this patient compared to both the other interferonopathy patients and healthy participants. In the ZNFX1-deficient patient, inflammatory pathways were activated, such as acute phase response signaling, and there was an enrichment of genes involved in macrophage activation signaling, neutrophil activation, and pathogen-induced cytokine storm signaling (Fig. 5A and Fig. S2A-D in the Online Resource). To further refine this comparison and assess the robustness of the findings, we performed a complementary pathway analysis comparing ZNFX1 vs interferonopathy patients in addition to the pooled contrast (ZNFX1 vs patients + controls) (Supplementary Fig. S4 in the Online Resource). The same inflammatory modules highlighted in Fig. 5A—acute phase response, HMGB1, macrophage activation, pyroptosis, Toll-like receptor, and neutrophil extracellular trap signaling—were consistently enriched across transcriptomic comparisons, supporting the robustness of the inflammatory program observed in the ZNFX1-deficient patient. The ZNFX1 vs interferonopathy comparison additionally revealed CGAS-STING, ERK/MAPK, RIPK1-mediated necrosis, and inflammasome signaling, indicating enhanced nucleic-acid sensing and stress-response pathways consistent with the patient’s hyper-inflammatory phenotype. These results indicated that other inflammatory signaling pathways were upregulated in addition to type I IFN signaling.

To identify potential cytokine(s) to target for therapeutic intervention, we first performed upstream regulator analysis using Ingenuity Pathway Analysis (IPA). The top 3 significant upstream regulator cytokines were interferon-gamma, TNF, and IL-1β (Fig. 5B). IL-6 was one of the top 10 most significant upstream regulators, and it was expressed more highly in EBV + B cells from the ZNFX1-deficient patient compared to healthy donors (Fig. S3A in the Online Resource). We next examined upstream cytokine regulators across the three transcriptomic contrasts by selecting the most significant regulators from each comparison (Supplementary Fig. S5 in the Online Resource). The ZNFX1 vs interferonopathy and ZNFX1 vs patients + controls contrasts produced highly concordant profiles, with IFN-γ, TNF, and IL-1β consistently predicted as activated, indicating that this cytokine activation profile remained consistent across the transcriptomic comparison strategies used in this study. Regulators associated with type I interferons (IFNA2, IFNB1, IFNA1/13, IFNL1) were predominant only when comparing ZNFX1 vs controls, as expected as this ISG signature is shared with the interferonopathy patients. We then measured plasma cytokine levels and found that the cytokines predicted in the upstream regulator analysis, that were detectable in the plasma were elevated in the patient compared to healthy participants (Fig. 5C and Fig. S3B-C in the Online Resource). We performed upstream regulator analysis on the over 90 cytokines measured in the plasma by Olink. Consistent with the transcriptomic data, the top 3 cytokine upstream regulators were still TNF, IL-1β, and interferon-gamma (Fig. S3D in the Online Resource).

## Clinical Comparisons of Type 1 Interferonopathies with *ZNFX1* Mutations

One study analyzed a group of 66 consecutive patients with undifferentiated autoinflammatory disease using cytokine profiles, ISGs and whole genome sequencing [1]. They compared patients with and without an interferon signature and noted significant clinical and laboratory differences (Table 2). We compared the clinical feature of our patient with their patients, and other patients with *ZNFX1* mutations [7]. All the patients presented at a very young age. Notably basal ganglia calcifications, interstitial lung disease, and transaminitis were common for those with classical interferonopathies and *ZNFX1* mutations. However, skin rashes, myositis, and vascular disease were not seen in the patients with *ZNFX1* mutations. Across the currently reported cases, patients with ZNFX1 deficiency appear to exhibit a higher frequency of kidney disease and MAS-like hyperinflammation compared with classical interferonopathies, although broader validation in larger cohorts is needed.

**Table 2:** Clinical manifestation of participants with and without Type I interferonopathies compared to participants with ZNFX1 mutations

	Interferon signature genes	Without interferon signature genes	Our patient	All ZNFX1 pooled patients^c^ (37)
Age of onset	0–15 months	1.5–57 months	2 months	10 days-72 months
Skin	+ + (Any rash)	+	-	+ 9/37
Neurological	+ + (Basal Ganglia Calcifications)	+	-	+ + 19/37
Pulmonary	+ + (ILD)	+	+	+ + 20/37
MSK	+ + (Myositis, Aseptic Osteomyelitis	+	-	- 1/37
Vascular	+ + (Arterial HTN)	+	-	- 0/37
Liver	+ + (Transaminitis)	+	+	+ + 29/37
sHLH/MAS^a^	+	+	+	+ + 18/37
Kidney	-	-	+	+ + 21/37
Mycobacterium/MSMD^b^			-	- 4/37

^a^MAS criteria shown in Table 1

^b^Three case series of patients with ZNFX1 reported 4 cases M. Kansasii after transplant, M.Bovis-BCG, M.Tuberculosis and M. Smegmatis [2, 7, 29]

^c^ZNFX1 Seven pooled case reports and case series totaling 37 patients and with our patient, total of 38 [2–7, 29]

## Clinical Comparisons of HLH with *ZNFX1* Mutations

Other studies suggest that participants with *ZNFX1* mutations should be added to the list of HLH-related mutations [7, 13]. Our patient presented similarly to patients with pHLH, with unremitting fevers, splenomegaly, hepatomegaly, high ferritin, cytopenias, and coagulopathy (Table 1). However, she developed TMA and later lung disease, neither being typical features of pHLH. TMA was described in 2 small cohorts of patients, 1 in children with systemic JIA, and another in a group of patients with refractory pHLH. Genetic testing was not available for the first cohort, and 12/23 in the second cohort had genetic pHLH [14, 15]. Interestingly, lung disease has been associated with MAS and Drug Reaction with Eosinophilia and Systemic Symptom (DRESS) syndrome in children with sJIA after treatment with IL-1 and IL-6 inhibition [16, 17].

## Discussion

Here we presented a child with a homozygous *ZNFX1* mutation who had repeated viral infections associated with multiorgan inflammation. The frameshift within the helicase domain yields a truncated ZNFX1 protein despite normal transcript levels, suggesting loss-of-function due to disruption of the helicase domain and loss of the C-terminal zinc-finger domains. Based on clinical similarity and elevated ISGs, seen in patients with interferonopathies, we hypothesized that this *ZNFX1* mutation, causes chronic unregulated interferon signaling, like SAVI, AGS, and PRAAS. However, we found that the ZNFX1-deficient patient had additional inflammatory cytokine pathways chronically upregulated, when compared to canonical type 1 interferonopathies. Furthermore, our patient and others with *ZNFX1* mutations have hyperinflammation akin to patients with MAS, with a distinguishing finding of TMA.

The upregulation of ISGs in our patient, was greater than healthy controls and comparable to participants with AGS. Clinically, our patient did not have the characteristic vasculopathies, skin and musculoskeletal manifestations seen in the classic interferonopathies. Similar to interferonopathy patients, low titer antibodies to dsDNA and ANCA were found in our ZNFX1 patient. Lymphopenia with low B and NK cell counts, and thrombocytopenia were notable in patients with elevated ISGs [18]. Similarly, our patient had lymphopenia early on, but her B and NK cell counts were normal initially, however with recurrent viral infections her B cells increased significantly. Unlike patients with interferonopathies, the IgG and IgM levels were always high until they were depleted with Rituximab. However, the NK cell counts started decreasing and persisted which may contribute to inefficient cytolytic activity, poor regulation of CD4 + and CD8^+^ T cells, and more time for a pathogen-initiated inflammatory cascade to release massive amounts of cytokines, that lead to MAS and sustained inflammation [19].

A marked difference in patients with *ZNFX1* mutations, was the presence of kidney disease, specifically TMA-like disease, and a MAS-like illness, that led to severe morbidity in our patient and in the larger cohort [7]. Although, ZNFX1 deficiency is associated with dysregulated ISG and an abnormal response to a viral trigger, other inflammatory cytokine pathways (e.g. IFN-γ, TNF, and IL-1β) were significantly upregulated compared to the classical interferonopathies, suggesting that ZNFX1 deficiency may represent a broader interferon-associated hyperinflammatory phenotype extending beyond classical type I interferonopathies. In initial data, we saw a transcriptomic signature featuring increased IL-6 signaling and complement activation. IL-6 is a pleiotropic cytokine that occurs in most inflammatory and infectious illnesses including systemic juvenile idiopathic arthritis, COVID-19 cytokine storm, and in MAS. In our patient, IL-6 signaling was a prominent part of her illness whether she was well or sick. Similarly, complement activation is a part of the non-specific inflammatory response, seen with IL-6 upregulation. Furthermore, upregulation of complement genes in response to interferon occurs in patients with TMA [20]. IFN-γ itself leads to complement activation via neutrophil extracellular traps (NETs) production [21]. Moreover, C5a is known to induce synthesis of both TNF-alpha and IL-1β, in monocytes and macrophages, which in turn reciprocally enhances the production of complement receptors in inflammatory cells [22]. Interestingly, eculizumab, anti-C5 treatment, given to our patient when she was acutely ill and for several months afterwards, played a role in partially modifying the inflammation as it did with other patients with pHLH and TMA [15].

Similar to our patient, some patients in the other ZNFX1 deficiency cohorts had TMA, and three were treated with eculizumab [7]. One patient in that cohort was also found to carry a homozygous deletion of the complement factor H-related proteins 1 and 3 (CFHR1/3) genes, with a kidney biopsy consistent with TMA [7] — a finding mirrored in our patient. Few similarities exist between the atypical hemolytic uremic syndrome (aHUS) clinical phenotype and ZNFX1 deficient patients; infections are triggers of TMA and 50–75% develop extrarenal disease [23–25]. Unlike ZNFX1 patients, the extrarenal disease with homozygous deletions of CFHR1/3 are mostly CNS, due to elevated blood pressures and stroke, gastrointestinal and cardiac, with only a small number developing pulmonary disease. Interestingly with eculizumab, both renal and extrarenal disease resolves. In our patient the renal disease resolved but the pulmonary disease worsened.

The CFHR1/3 deletion warrants careful consideration. The strongest and most well-established pathogenic mechanism linking this deletion to aHUS operates through the DEAP-HUS pathway, wherein the deletion creates immunological permissiveness for anti–Factor H (anti-FH) autoantibody formation [24, 26]. In our patient, anti-FH antibodies were specifically tested and were negative, making this mechanism unlikely. Meta-analytic data further support this interpretation: CFHR1/3 deletion combined with anti-FH antibodies confers a markedly elevated aHUS risk (OR 11.75, 95% CI 4.53–30.44), whereas CFHR1/3 deletion alone, in the antibody-negative setting, is not significantly associated with aHUS risk (OR 1.32, 95% CI 0.50–3.50; p = 0.56) [27]. Consistent with these data, the homozygous CFHR1/3 deletion is a common population-level polymorphism, with homozygosity rates of approximately 8–10% globally — ranging from ~ 2–5% in European to as high as 54.7% in some African populations — and the vast majority of carriers never develop aHUS [28].

From a clinical standpoint, when our patient developed pulmonary hemorrhage and a flare in inflammatory markers, comprehensive functional complement assays (AH50, CH50, C3, and C4) were repeated alongside eculizumab plasma levels. Both the functional complement panel and drug trough levels were confirmed to be within therapeutic range, with no biochemical evidence of ongoing complement dysregulation. Despite adequate and confirmed complement blockade, the patient's multisystem inflammation continued to progress — not through a recurrent TMA picture, but through a non-complement-mediated inflammatory process. This clinical trajectory is fundamentally inconsistent with primary complement-driven disease as the sole or dominant pathological mechanism.

We therefore propose that the TMA observed in our patient most likely represented secondary complement dysregulation occurring in the context of the underlying ZNFX1-associated interferonopathy, rather than a primary complement defect. The transient benefit of eculizumab likely reflected attenuation of an already-activated complement cascade rather than correction of a primary pathological process. That said, we acknowledge that the CFHR1/3 deletion may serve as a phenotypic modifier — potentially lowering the threshold for complement activation under the hyperinflammatory conditions of ZNFX1 deficiency — and this possibility deserves further investigation.

Biallelic *ZNFX1* loss-of-function mutations have been found to result in a multi-organ inflammatory disease with susceptibility to viral infections or mycobacterial disease [29]. A previous study suggested that *ZNFX1* mutations should be added to the list of mutations associated with pHLH after an initiating viral trigger [13]. Typically, pHLH/MAS has occurred in patients with abnormal lymphocyte cytotoxicity, dysregulated inflammasome, impaired viral clearance, and immune dysregulation. In ZNFX1-deficient patients, the viral clearance is impaired, and the half-life of ISGs is increased following extensive stimulation (24 h) with intracellular DNA which can trigger the MAS response [7]. Supportive evidence in a child with a *ZNFX1* mutation and suspected MAS, demonstrated higher expression of IL-1β in unstimulated and stimulated neutrophils, and notably abnormally elevated naïve B cells and low memory B cells [30]. The precise mechanism of how the chronic interferon signaling in ZNFX1 deficient patients, leads to MAS is yet to be fully elucidated, however, models of MAS and ZNFX1 deficient mice may help our understanding. The best-characterized example of viral-mediated MAS is in X-linked lymphoproliferative syndrome 1 (XLP1), where EBV is not cleared due to poorly functioning NK cells and cytolytic T cells which results in further lymphocyte stimulation and cytokine production, leading to tissue macrophage activation [31, 32]. The activated macrophages hemophagocytose and produce a cascade of cytokines. Another example of an autoinflammatory disease developing MAS is IL-18PAP-MAS presenting with recurrent MAS, pulmonary alveolar proteinosis (PAP) and nail clubbing, with very high levels of serum IL-18, and CXCL9 that correlated with ISGs [1]. Similarly, children with systemic JIA/Still’s have high levels of IL-18 [33], a subset develop lung disease, and those who develop MAS have increased levels of IFN-γ and IFN induced chemokines. Phenotypically, ZNFX1 deficiency resembles both groups in a few ways, as they develop aggressive lung disease, with similar histologic features [16, 34–36], develop MAS and present early in life. ZNFX1 deficiency differs in the lack of rashes or musculoskeletal inflammation, attenuated IL-18 expression, recurrent viral infections, and strongly express type II IFN pathways. Adding to this landscape of interferonopathy-mediated hyperinflammatory disease, a subset within a cohort of treatment refractory Still’s disease (sJIA, Adult onset Still’s disease and Still’s like) had a prominent Type I IFN which was strongly associated with lung disease [37]. Thus ZNFX1 deficient patients who have lung disease may lie on a Type 1 interferon spectrum of other monogenic interferonopathies, IL-18/PAP-MAS, and these Still’s lung patients.

Another association between systemic JIA/Still’s lung disease and MAS, is the association with DRESS syndrome and IL1/IL6 inhibition, and of HLA-DRB1*15 alleles. Interestingly we found HLA-DRB1*15:02 present in our patient. Given that this is still being debated and actively investigated, with cohorts showing equal numbers of these alleles in systemic JIA patients with and without lung disease, we speculate that the presence of this allele in our ZNFX1 deficient patient may be of interest and needs to be investigated further. It is possible that this allele is present in other systemic inflammatory diseases presenting with MAS and lung disease [36, 38].

ISGs have transcription factors in their regulatory domains that are activated by recombinant IL-18 which may explain how ISGs are expressed in patients with IL-18PAP-MAS, and systemic JIA, and how high levels of IL-18 induce the production of IFN-γ which is critical in the pathogenesis of MAS [32, 39]. The common cytokine among these disease models is IFN-γ which perpetuates the ongoing hyperinflammation. In our patient, Olink cytokine analysis showed many abnormal cytokines typically seen in MAS such as IL-1β, IL-6, IL-18, TNF, CXCL9 and IFN-γ [40]. A recent study used the ZNFX1 mouse model postulated a mechanistic link, where wild type ZNFX1 functions as an inhibitor of the NLRP3 inflammasome activation [41]. Thus, mutations in *ZNFX1*, together with chronic interferon signaling, and poor inhibition on the inflammasome, lead to overexpression of IL-1β, IL-18, more IFN-γ and macrophage activation, serving as a plausible mechanism for patients with *ZNFX1* mutations to develop MAS.

Another 3 cases of patients with *ZNFX1* mutations had a different phenotype with susceptibility to mycobacterial infection similar to patients with Mendelian Susceptibility to Mycobacterial Diseases (MSMD) [29]. However, IFN-γ signaling was intact and normal in these patients going against the idea of the typical pathway of MSMD’s affecting the response or production of IFN-γ [29]. Interestingly in the cohort of pooled patients with *ZNFX1* mutations, only one patient had a post lung transplant mycobacterium infection, suggesting a variable phenotype potentially depending on the variant location and function [2, 7]. Overtime as we identify more patients, we may confirm whether *ZNFX1* mutations confer a higher risk of mycobacterial infections. Supporting this possibility, a recent report showed that bone marrow–derived macrophages from ZNFX1^⁻/⁻^ mice infected with *Mycobacterium tuberculosis* exhibited impaired autophagy and defective bacterial clearance, suggesting a mechanism that may contribute to susceptibility in ZNFX1-deficient patients [42].

One significant limitation of our study is that there are 38 cases (including our patient) described so far with variability in presentation which will require larger cohorts to validate our conclusion that this is a distinct disease. Due to the limited number of reported ZNFX1-deficient cases and the use of longitudinal samples from a single deeply profiled index patient, comparisons with interferonopathies and MAS-associated disorders remain exploratory, descriptive, and hypothesis-generating, highlighting the need for larger collaborative studies to validate and quantitatively refine these findings. Another limitation is the absence of a fully established mechanism for the TMA that occurs in many ZNFX1-deficient patients. The homozygous CFHR1/3 deletion found in our patient — and in at least one other ZNFX1-deficient patient [7] — is an important consideration. CFHR1/3 deletion may nonetheless act as a susceptibility modifier, potentially amplifying complement activation under the hyperinflammatory physiological state imposed by ZNFX1 deficiency. Prospective functional complement studies in larger ZNFX1 cohorts — with systematic testing for anti-FH antibodies and careful correlation with TMA occurrence — will be essential to resolve this question.

Another limitation of our study is that the transcriptomic and plasma proteomic data is from a single ZNFX1-deficient patient. However, the whole blood transcriptomics data is from 3 different blood draws each taken approximately a month or more apart, and the plasma proteomic data is from 2 different blood draws taken more than 3 months apart in the absence of any overt infection. Since these data were generated from multiple time points, this indicates that the inflammatory signatures identified were chronic and persistent in the patient, supporting the significance of these findings.

Our goal in interrogating our patient’s genetic mutation, and its functional implications was to find a treatment. Using eculizumab helped with TMA and MAS early on, but did not stop the progressive lung and hepatic disease. Broad immunosuppression with Rituximab and cyclophosphamide, prior to knowing her genetic mutation, proved detrimental and underlined the need for targeted therapy. In the large patient cohort, all patients were treated with corticosteroids and a variety of immunosuppressants including anti-IL-1β, JAK inhibitor, etoposide, and eculizumab which were partially successful with short-lived benefit. One patient had hematopoietic stem cell transplant (HSCT) and remains alive [7]. Given the information from our transcriptomic data, IL-1β inhibition, IL-6 inhibition, anti-IFN-γ monoclonal antibody, and IL-18 binding protein (IL-18BP) are treatments that deserve further investigation in larger cohorts [39, 40]. The data from Gloude and colleagues in their cohort of patients with pHLH and TMA, suggest there is a “IFN-γ-complement loop” that causes relentless inflammation with ensuing organ damage, and in their patients, a treatment combination of anti-IFN-γ and eculizumab was successful [15]. Furthermore, the use of anti-IFN-γ interestingly has been used as HSCT conditioning to promote engraftment because high IFN-γ levels may interfere with stem cell function and increase apoptosis [43]. Thus, in addition to first line treatment, it may serve a dual benefit in preparing the patient for successful HSCT.

In conclusion, ZNFX1 deficiency is associated with severe early-onset multisystem inflammatory disease whose presentation may include co-occurring MAS and TMA. The disease process may involve persistent ISG upregulation following viral triggers together with dysregulated inflammasome activation and exuberant cytokine production. Given that only a few cases have been published so far, there is a need for a high degree of suspicion to consider this diagnosis in children with similar findings, and to characterize their immune dysfunction in order to understand the variability in clinical presentation. Given the significant morbidity and mortality, early evaluation of transcriptomics and targeted treatment is necessary with cytokine modulating therapy to curb the early inflammation and subsequent damage, while pursuing early HSCT.

## Supplementary Information

Below is the link to the electronic supplementary material.

Supplementary file1 (PDF 34741 KB)

Supplementary file2 (PDF 66760 KB)

Supplementary file3 (PDF 15080 KB)

Supplementary file4 (PDF 13 KB)

Supplementary file5 (PDF 11 KB)

Supplementary file6 (XLSX 13 KB)

Supplementary file7 (XLSX 11739 KB)

Supplementary file8 (XLSX 23 KB)

Supplementary file9 (DOCX 27 KB)

## Data Availability

The datasets generated during and/or analyzed during the current study are available from the corresponding author on reasonable request.

## Abbreviations

MAVS: Mitochondrial antiviral signaling protein
ISGs: Interferon-stimulated genes
MAS: Macrophage activation syndrome
TNF: Tumor necrosis factor
TMA: Thrombotic microangiopathy
IFN: Interferon
JAK-STAT: Janus kinase-signal transducer and activator of transcription
SAVI: STING-associated vasculopathy with onset in infancy
PRAAS: Proteasome- Associated Autoinflammatory Syndromes
AGS: Aicardi Goutières syndrome
sHLH: Secondary hemophagocytic lymphohistiocytosis
pHLH: Primary Hemophagocytic lymphohistiocytosis
aHUS: Atypical hemolytic uremic syndrome
IVIG: Intravenous immunoglobulin
AH50: Alternative complement pathway activity
CFHR1 and CFHR3: Complement factor H related protein 1 and 3
WES: Whole exome sequencing
BAL: Broncho-alveolar lavage
ANA: Antinuclear antibody
Anti-GBM: Anti-glomerular basement membrane
ANCA: Antineutrophil cytoplasmic antibody
dsDNA: Double-stranded DNA
CMV: Cytomegalovirus
HSV: Herpes simplex virus
LSS: Lymphocyte subsets
DRESS: Drug Reaction with Eosinophilia and Systemic Symptom
