# Supplementary material for: Unraveling Immune Dysregulation in ZNFX1 Deficiency: From Interferonopathies to Macrophage Activation Syndrome
Source: J Clin Immunol. 2026 Aug 6;46(1):100. doi: 10.1007/s10875-026-02056-4 (PMC13727932; doi:10.1007/s10875-026-02056-4)
Supplement: Supplementary file 1 [file 10875_2026_2056_MOESM1_ESM.pdf]

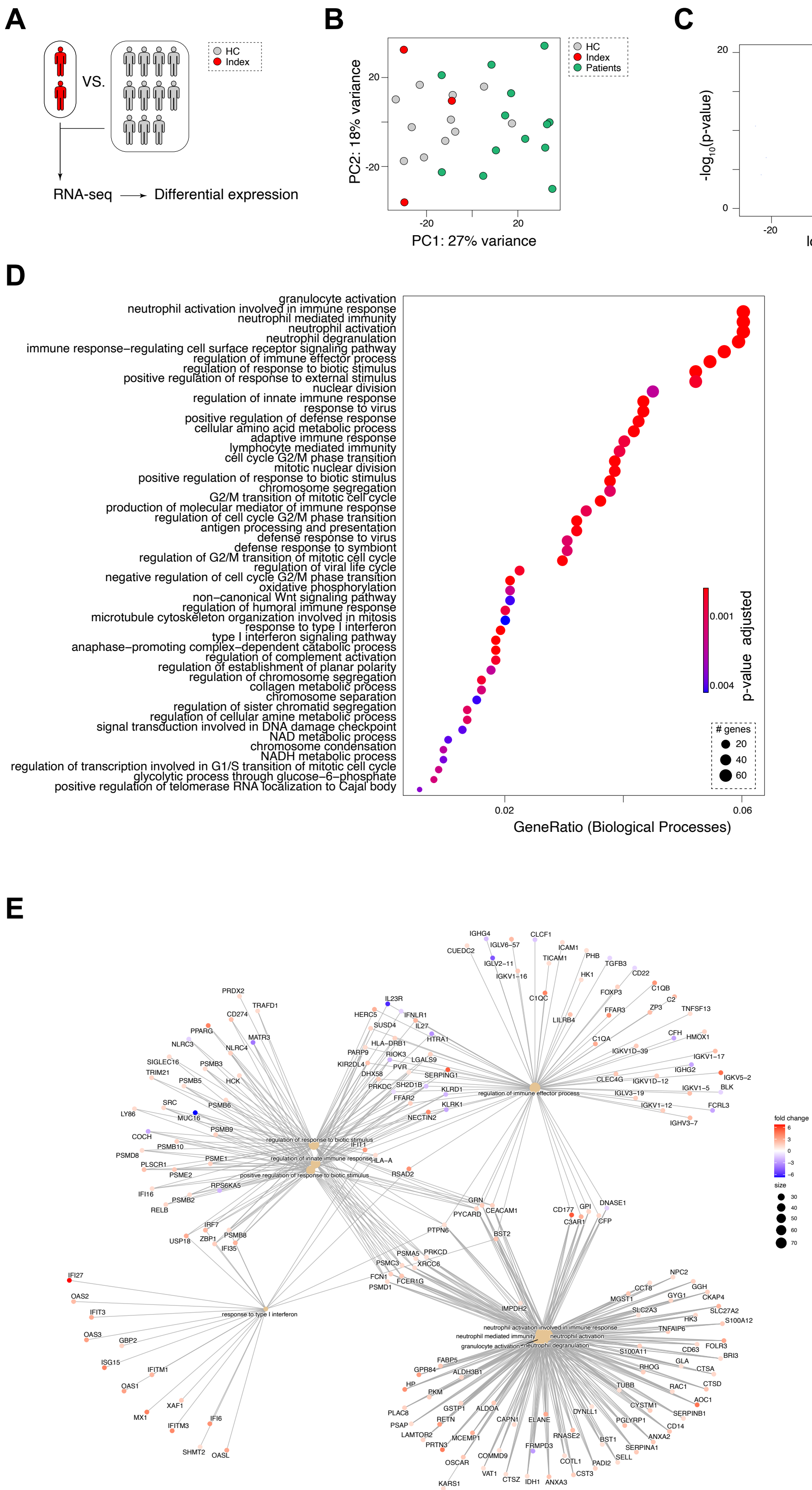

A
HC
Index
VS.
RNA-seq
Differential expression
B
HC
Index
Patients
PC2: 18% variance
PC1: 27% variance
20
-20
-20
20
C
-log10(p-value)
log2 fold change
20
15
10
5
0
-20
20
D
granulocyte activation
neutrophil activation involved in immune response
neutrophil mediated immunity
neutrophil activation
neutrophil degranulation
immune response−regulating cell surface receptor signaling pathway
regulation of immune effector process
regulation of response to biotic stimulus
positive regulation of response to external stimulus
nuclear division
regulation of innate immune response
response to virus
positive regulation of defense response
cellular amino acid metabolic process
adaptive immune response
lymphocyte mediated immunity
cell cycle G2/M phase transition
mitotic nuclear division
positive regulation of response to biotic stimulus
chromosome segregation
G2/M transition of mitotic cell cycle
production of molecular mediator of immune response
regulation of cell cycle G2/M phase transition
antigen processing and presentation
defense response to virus
defense response to symbiont
regulation of G2/M transition of mitotic cell cycle
regulation of viral life cycle
negative regulation of cell cycle G2/M phase transition
oxidative phosphorylation
non−canonical Wnt signaling pathway
regulation of humoral immune response
microtubule cytoskeleton organization involved in mitosis
response to type I interferon
type I interferon signaling pathway
anaphase−promoting complex−dependent catabolic process
regulation of complement activation
regulation of establishment of planar polarity
regulation of chromosome segregation
collagen metabolic process
chromosome separation
regulation of sister chromatid segregation
regulation of cellular amine metabolic process
signal transduction involved in DNA damage checkpoint
NAD metabolic process
chromosome condensation
NADH metabolic process
regulation of transcription involved in G1/S transition of mitotic cell cycle
glycolytic process through glucose−6−phosphate
positive regulation of telomerase RNA localization to Cajal body
0.02
0.04
0.06
GeneRatio (Biological Processes)
p-value adjusted
0.001
0.004
genes
20
40
60
E
regulation of immune effector process
regulation of response to biotic stimulus
regulation of innate immune response
positive regulation of response to biotic stimulus
response to type I interferon
neutrophil activation involved in immune response
neutrophil mediated immunity
neutrophil activation
granulocyte activation
neutrophil degranulation
fold change
6
3
0
-3
-6
size
30
40
50
60
70
