# Supplementary material for: Unraveling Immune Dysregulation in ZNFX1 Deficiency: From Interferonopathies to Macrophage Activation Syndrome
Source: J Clin Immunol. 2026 Aug 6;46(1):100. doi: 10.1007/s10875-026-02056-4 (PMC13727932; doi:10.1007/s10875-026-02056-4)
Supplement: Supplementary file 2 [file 10875_2026_2056_MOESM2_ESM.pdf]

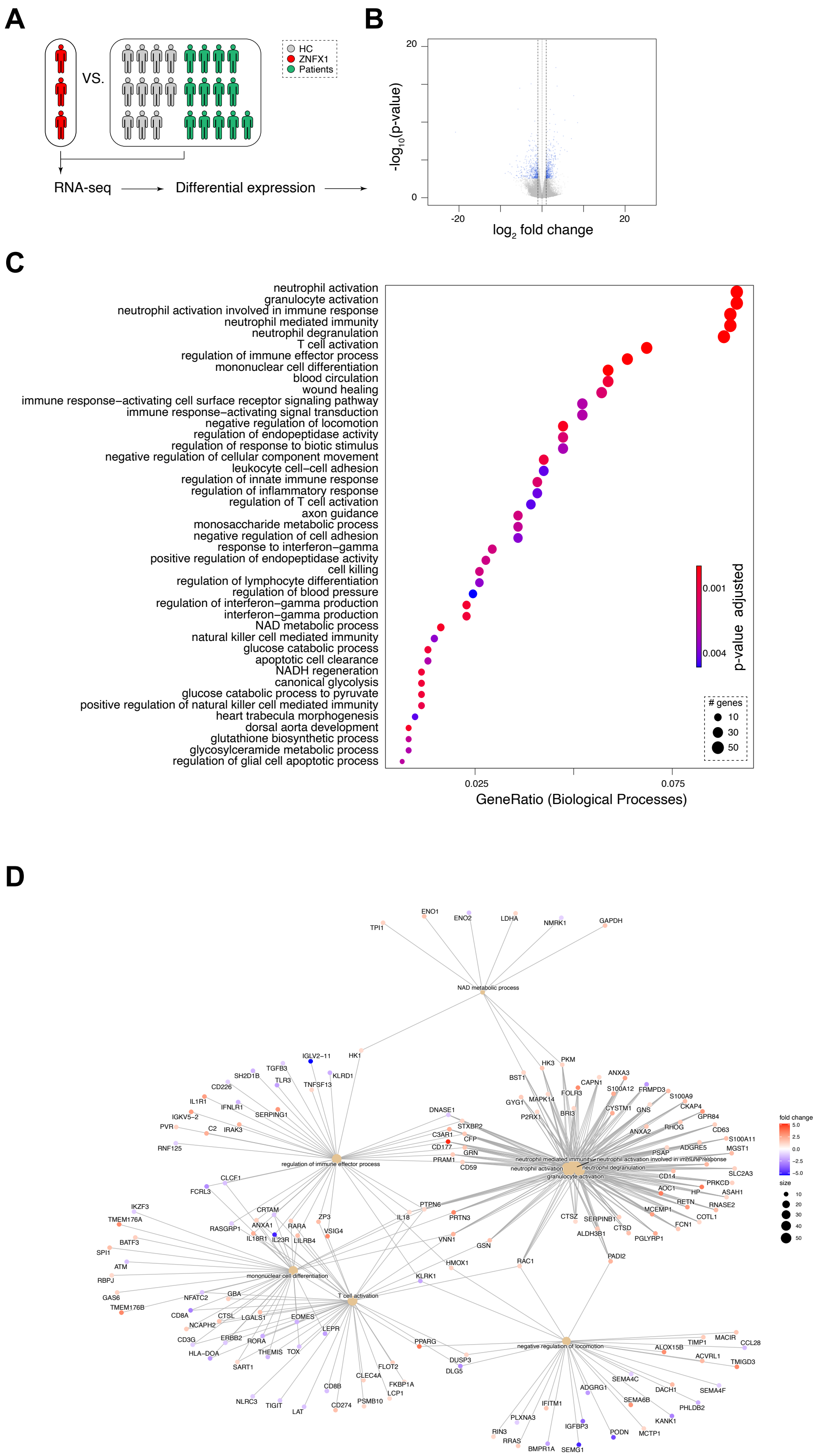
A
VS.
HC
ZNFX1
Patients
RNA-seq
Differential expression
B
20
0
-20
20
$-\log_{10}$(p-value)
$\log_2$ fold change
C
neutrophil activation
granulocyte activation
neutrophil activation involved in immune response
neutrophil mediated immunity
neutrophil degranulation
T cell activation
regulation of immune effector process
mononuclear cell differentiation
blood circulation
wound healing
immune response−activating cell surface receptor signaling pathway
immune response−activating signal transduction
negative regulation of locomotion
regulation of endopeptidase activity
regulation of response to biotic stimulus
negative regulation of cellular component movement
leukocyte cell−cell adhesion
regulation of innate immune response
regulation of inflammatory response
regulation of T cell activation
axon guidance
monosaccharide metabolic process
negative regulation of cell adhesion
response to interferon−gamma
positive regulation of endopeptidase activity
cell killing
regulation of lymphocyte differentiation
regulation of blood pressure
regulation of interferon−gamma production
interferon−gamma production
NAD metabolic process
natural killer cell mediated immunity
glucose catabolic process
apoptotic cell clearance
NADH regeneration
canonical glycolysis
glucose catabolic process to pyruvate
positive regulation of natural killer cell mediated immunity
heart trabecula morphogenesis
dorsal aorta development
glutathione biosynthetic process
glycosylceramide metabolic process
regulation of glial cell apoptotic process
0.025
0.075
GeneRatio (Biological Processes)
p-value adjusted
0.001
0.004
genes
10
30
50
D
NAD metabolic process
regulation of immune effector process
neutrophil mediated immunity
neutrophil activation involved in immune response
neutrophil activation
neutrophil degranulation
granulocyte activation
mononuclear cell differentiation
T cell activation
negative regulation of locomotion
fold change
5.0
2.5
0.0
−2.5
−5.0
size
10
20
30
40
50
