# Supplementary material for: Unraveling Immune Dysregulation in ZNFX1 Deficiency: From Interferonopathies to Macrophage Activation Syndrome
Source: J Clin Immunol. 2026 Aug 6;46(1):100. doi: 10.1007/s10875-026-02056-4 (PMC13727932; doi:10.1007/s10875-026-02056-4)
Supplement: Supplementary file 3 [file 10875_2026_2056_MOESM3_ESM.pdf]

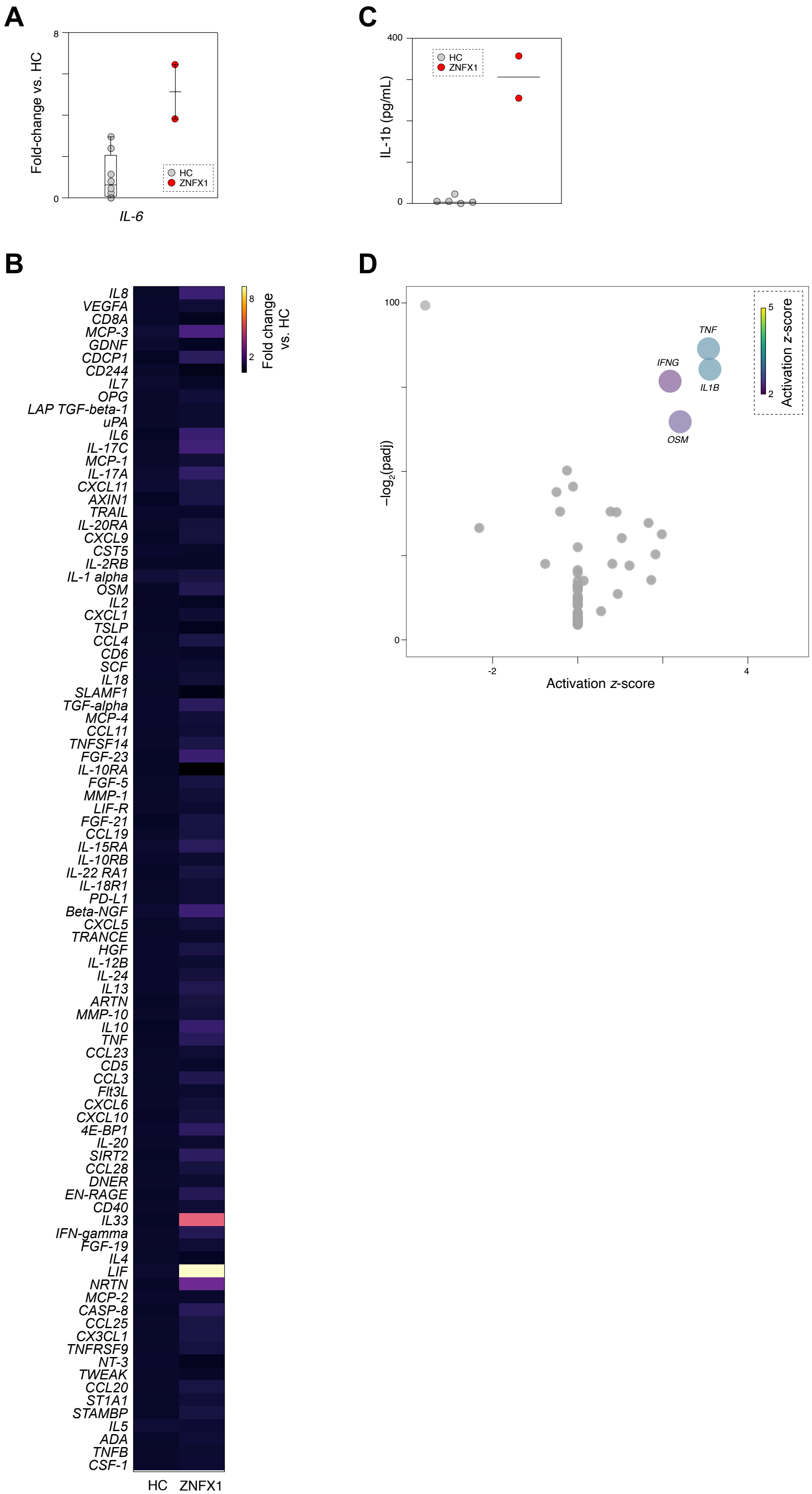
A
Fold-change vs. HC
8
0
HC
ZNFX1
IL-6
C
400
0
IL-1b (pg/mL)
HC
ZNFX1
B
IL8
VEGFA
CD8A
MCP-3
GDNF
CDCP1
CD244
IL7
OPG
LAP TGF-beta-1
uPA
IL6
IL-17C
MCP-1
IL-17A
CXCL11
AXIN1
TRAIL
IL-20RA
CXCL9
CST5
IL-2RB
IL-1 alpha
OSM
IL2
CXCL1
TSLP
CCL4
CD6
SCF
IL18
SLAMF1
TGF-alpha
MCP-4
CCL11
TNFSF14
FGF-23
IL-10RA
FGF-5
MMP-1
LIF-R
FGF-21
CCL19
IL-15RA
IL-10RB
IL-22 RA1
IL-18R1
PD-L1
Beta-NGF
CXCL5
TRANCE
HGF
IL-12B
IL-24
IL13
ARTN
MMP-10
IL10
TNF
CCL23
CD5
CCL3
Flt3L
CXCL6
CXCL10
4E-BP1
IL-20
SIRT2
CCL28
DNER
EN-RAGE
CD40
IL33
IFN-gamma
FGF-19
IL4
LIF
NRTN
MCP-2
CASP-8
CCL25
CX3CL1
TNFRSF9
NT-3
TWEAK
CCL20
ST1A1
STAMBP
IL5
ADA
TNFB
CSF-1
HC
ZNFX1
8
2
Fold change vs. HC
D
100
0
-log2(padj)
-2
4
Activation z-score
TNF
IL1B
IFNG
OSM
5
2
Activation z-score
