# Supplementary material for: Unraveling Immune Dysregulation in ZNFX1 Deficiency: From Interferonopathies to Macrophage Activation Syndrome
Source: J Clin Immunol. 2026 Aug 6;46(1):100. doi: 10.1007/s10875-026-02056-4 (PMC13727932; doi:10.1007/s10875-026-02056-4)
Supplement: Supplementary file 4 [file 10875_2026_2056_MOESM4_ESM.pdf]

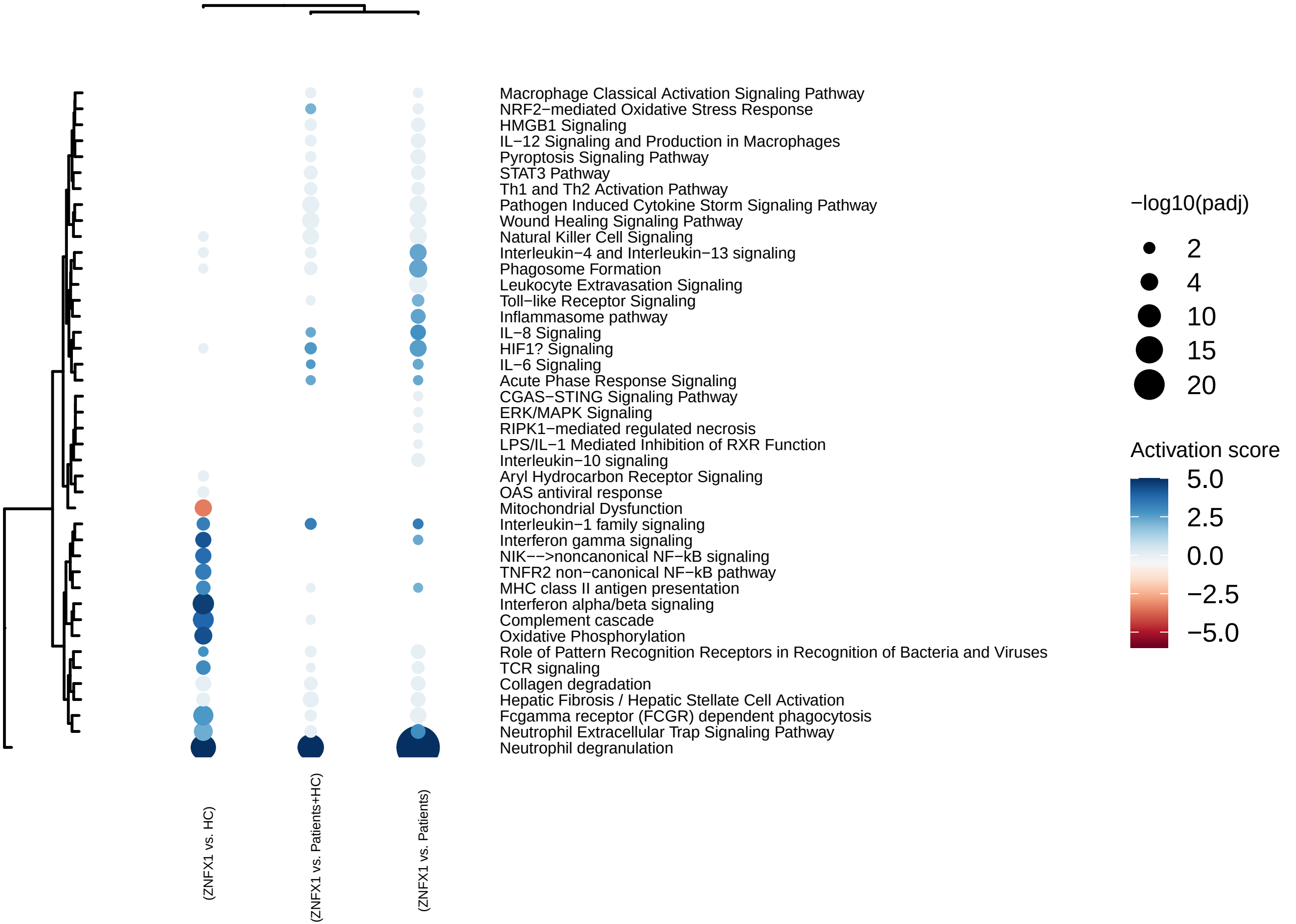

Macrophage Classical Activation Signaling Pathway
NRF2−mediated Oxidative Stress Response
HMGB1 Signaling
IL−12 Signaling and Production in Macrophages
Pyroptosis Signaling Pathway
STAT3 Pathway
Th1 and Th2 Activation Pathway
Pathogen Induced Cytokine Storm Signaling Pathway
Wound Healing Signaling Pathway
Natural Killer Cell Signaling
Interleukin−4 and Interleukin−13 signaling
Phagosome Formation
Leukocyte Extravasation Signaling
Toll−like Receptor Signaling
Inflammasome pathway
IL−8 Signaling
HIF1? Signaling
IL−6 Signaling
Acute Phase Response Signaling
CGAS−STING Signaling Pathway
ERK/MAPK Signaling
RIPK1−mediated regulated necrosis
LPS/IL−1 Mediated Inhibition of RXR Function
Interleukin−10 signaling
Aryl Hydrocarbon Receptor Signaling
OAS antiviral response
Mitochondrial Dysfunction
Interleukin−1 family signaling
Interferon gamma signaling
NIK−−>noncanonical NF−kB signaling
TNFR2 non−canonical NF−kB pathway
MHC class II antigen presentation
Interferon alpha/beta signaling
Complement cascade
Oxidative Phosphorylation
Role of Pattern Recognition Receptors in Recognition of Bacteria and Viruses
TCR signaling
Collagen degradation
Hepatic Fibrosis / Hepatic Stellate Cell Activation
Fcgamma receptor (FCGR) dependent phagocytosis
Neutrophil Extracellular Trap Signaling Pathway
Neutrophil degranulation
(ZNFX1 vs. HC)
(ZNFX1 vs. Patients+HC)
(ZNFX1 vs. Patients)
−log10(padj)
2
4
10
15
20
Activation score
5.0
2.5
0.0
−2.5
−5.0
