# Supplementary material for: Unraveling Immune Dysregulation in ZNFX1 Deficiency: From Interferonopathies to Macrophage Activation Syndrome
Source: J Clin Immunol. 2026 Aug 6;46(1):100. doi: 10.1007/s10875-026-02056-4 (PMC13727932; doi:10.1007/s10875-026-02056-4)
Supplement: Supplementary file 5 [file 10875_2026_2056_MOESM5_ESM.pdf]

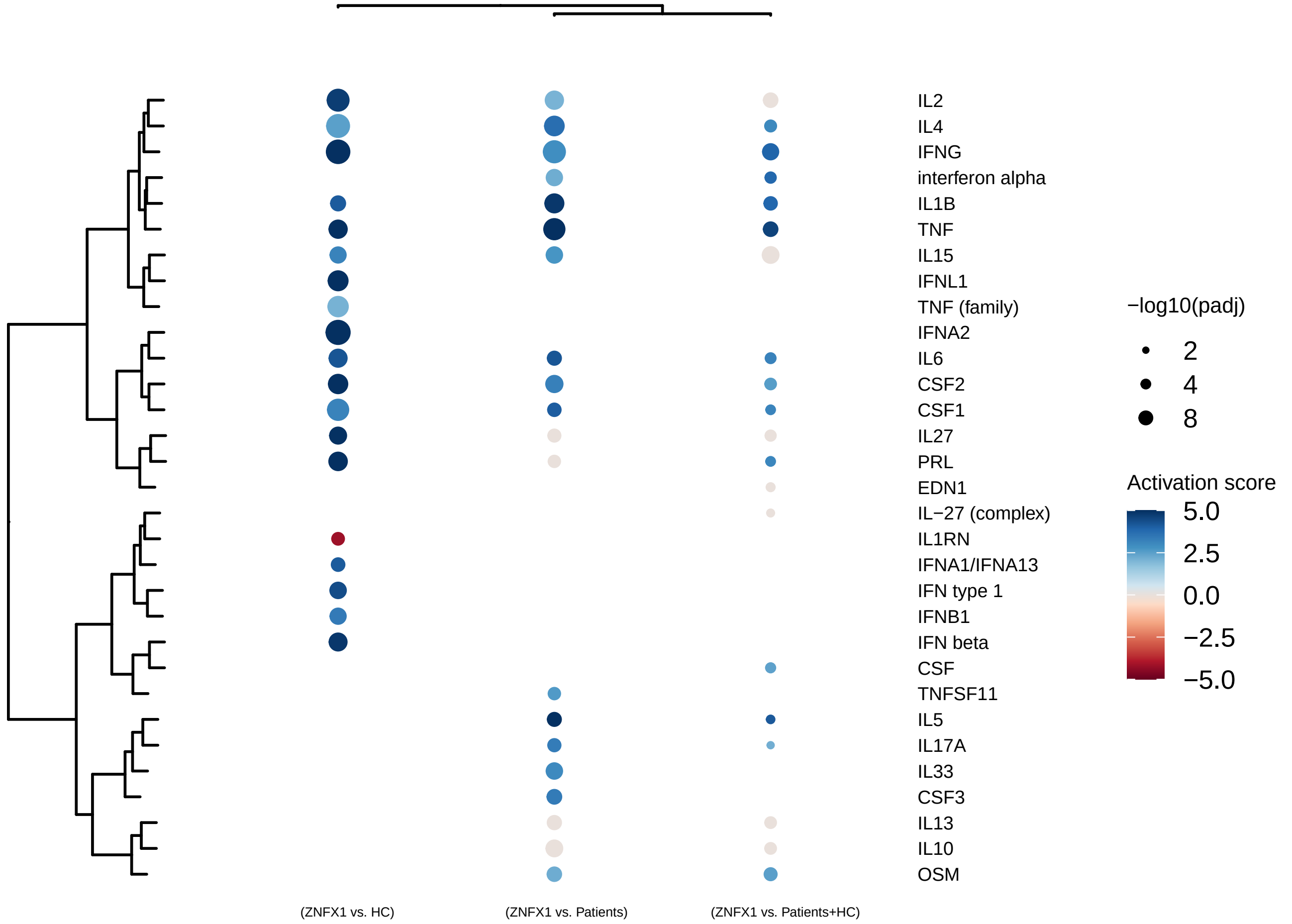
IL2
IL4
IFNG
interferon alpha
IL1B
TNF
IL15
IFNL1
TNF (family)
IFNA2
IL6
CSF2
CSF1
IL27
PRL
EDN1
IL−27 (complex)
IL1RN
IFNA1/IFNA13
IFN type 1
IFNB1
IFN beta
CSF
TNFSF11
IL5
IL17A
IL33
CSF3
IL13
IL10
OSM
−log10(padj)
2
4
8
Activation score
5.0
2.5
0.0
−2.5
−5.0
(ZNFX1 vs. HC)
(ZNFX1 vs. Patients)
(ZNFX1 vs. Patients+HC)
