# Supplementary material for: Unraveling Immune Dysregulation in ZNFX1 Deficiency: From Interferonopathies to Macrophage Activation Syndrome
Source: J Clin Immunol. 2026 Aug 6;46(1):100. doi: 10.1007/s10875-026-02056-4 (PMC13727932; doi:10.1007/s10875-026-02056-4)
Supplement: Supplementary file 6 [file 10875_2026_2056_MOESM9_ESM.docx]

**Supplementary Section**

**Method**

**HLA Genotyping**

HLA genotyping of the proband WGS data was done using HLA-HD v 1.7.1 [1]. This involved extraction of reads overlapping the whole MHC genomic region, in addition to the unmapped reads from BAM file, which were then converted to Fastq file format and run through the typing process to get HLA genotypes at the maximum possible resolution.

**Western blot analysis of ZNFX1 expression**

EBV+B were harvested, washed 1X with PBS and lysed with RIPA buffer containing 1mM PMSF and 10 mM NEM, then boiled at 90ºC for 5 minutes in 1× Laemmlli buffer. Eluates then were loaded onto 4%–15% TGX gels (Bio-Rad, Hercules, CA). Proteins were then transferred onto nitrocellulose membrane and blotted with anti-ZNFX1 (abcam, ab179452) and anti β-Actin (proteintech, HRP-60008) antibodies.

**Real-Time Quantitative PCR (RT-qPCR)**

RNA was extracted from EBV+B cells using the RNeasy Mini kit (Qiagen) as per manufacturer’s instructions, cDNA was synthesized using SuperScript VILO kit (Invitrogen, 11754-050) and qPCR was performed using the Fast SYBR Green Master Mix (Applied Biosynthesis, 4385612). The Fast 96 well plate was read using QuantStudio™ 12K Flex System (Thermo Fisher). All reactions were run for 35 cycles in duplicate, and the comparative delta delta CT (∆∆Ct ) method was used to analyze the data with RPLPO as the housekeeping gene control. The following primers were used to quantify transcripts from genes of interest: RPLPO 5’-AGCCCAGAACACTGGTCTC-3’ and 5’-ACTCAGGATTTCAATGGTGCC-3’; IL-6	5’-ACTCACCTCTTCAGAACGAATTG-3’ and 5’-CCATCTTTGGAAGGTTCAGGTTG-3’; ZNFX1 5’-AAATCAGGCCAATAACCCACC-3’ and 5’-GGCCCTAAATCTCTCTTCCCT-3’

**Supplementary Section**

**Figures**

**Figure S1** Differential expression analysis of index patient versus healthy controls (HC)

A Schematic of the whole blood mRNA-seq samples selected for the comparison in the differential expression analysis. Two independent blood draws taken approximately a month apart from the index patient were used for the differential expression analysis

B Principal-component analysis (PCA) of the 1000 most expressed genes across all RNA-seq samples from this study. Patients (green) indicates the other unrelated patients with known interferonopathies

C Volcano plot of adjusted p-values vs. log2 fold change of mean expression (index case vs healthy controls). Blue dots mark significant differentially expressed (DE) genes (|log2FC| ≥ 1, adj p < 0.05), gray dots indicate non-DE genes, and red dots highlight DE genes from the 28 IFN-response gene list. Positive log2FC indicates upregulation in the index patient

D Dot plot of the top 50 significantly enriched Gene Ontology (GO) Biological Process terms identified by over-representation analysis of differentially expressed genes (index patient vs. healthy controls). Gene ratio indicates the proportion of significant genes associated with each GO term. Dot size reflects the number of enriched genes, while dot color corresponds to the adjusted p-value. This analysis highlights dominant antiviral, interferon-mediated, and innate-immune activation programs in the index patient.

E Category net plot showing the connections between the top 10 most significantly enriched Gene Ontology Biological Process terms and their associated differentially expressed genes (index patient vs. healthy controls). Edge links indicate gene–term associations. Gene nodes are colored by log₂ fold change, and GO term node size reflects the number of enriched genes per term.

This network illustrates that several key antiviral and inflammatory genes—such as ISGs, OAS family members, IFITs, neutrophil-related effectors, and antigen-presentation genes—are shared across multiple enriched GO terms, revealing tightly coordinated activation of interferon signaling, innate immune defense, and neutrophil-mediated inflammatory pathways in the index patient.

**Figure S2** Differential expression analysis of ZNFX1 patient versus the other interferonopathy patients and healthy controls (P+HC)

A Schematic of whole blood mRNA-seq samples selected for the comparison in the differential expression analysis. Three independent blood draws from the patient taken at least 1 month apart were used for the differential expression analysis. Patients (in green) represents those with known interferonopathies

B Volcano plot summarizing differential expression analysis. Blue dots mark significant DE genes (|log2FC| ≥ 1, adj p < 0.05), while gray dots indicate non-DE genes. The x-axis shows log2 fold change (ZNFX1 vs. P+HC), and the y-axis represents log10-transformed FDR-adjusted p-values

C Dot plot of the top 50 significantly enriched Gene Ontology Biological Process terms from over-representation analysis of differentially expressed genes (ZNFX1 vs. patients + healthy controls). Gene ratio represents the proportion of significant genes per term. Dot size indicates the number of enriched genes per term, and dot color reflects the adjusted p-value. This analysis highlights strong enrichment of neutrophil activation, granulocyte degranulation, interferon-γ responses, and broader innate immune effector functions that collectively characterize the hyper-inflammatory transcriptional profile of the ZNFX1-deficient patient.

D Category net plot illustrating the relationship between the top 10 most significantly enriched Gene Ontology Biological Process terms and their associated differentially expressed genes (ZNFX1 vs patients + healthy controls). Each GO term is represented as a central node, with edges connecting it to its contributing genes. Node size reflects the number of enriched genes within each term, while gene node color denotes log₂ fold change direction and magnitude.

This network highlights extensive overlap among genes driving multiple biological processes, particularly those involved in neutrophil activation, immune effector regulation, and cellular stress responses, underscoring the coordinated inflammatory program in the ZNFX1 deficient patient.

**Figure S3** Inflammatory cytokines upregulated in the patient compared to healthy donors.

A IL-6 mRNA expression levels from EBV+B lymphocytes shown as fold-change of IL-6 transcript levels from qPCR of patient versus healthy controls (HC)

B Heatmap displaying relative cytokine levels as measured by OLINK experiment. Protein levels are represented as fold change of ZNFX1 patient levels over HC

C IL-1β levels as measured by ELISA

D Dot plot highlighting significant cytokine upstream regulators predicted from the plasma proteomic profile (~90 cytokines measured by Olink) in the ZNFX1-deficient patient compared with healthy controls. Dot size reflects statistical significance (–log₂ adjusted p-value), and dot color denotes the predicted activation z-score.

IFN-γ, TNF, and IL-1β emerge as the dominant activated cytokine regulators, consistent with the hyper-inflammatory signature identified in the index patient.

**Figure S4** Comparative pathway enrichment analysis across three RNA-seq contrasts

Dot plot showing canonical pathways significantly enriched (adjusted p < 0.05) across three contrasts: ZNFX1 vs. healthy controls, ZNFX1 vs. interferonopathy patients, and ZNFX1 vs. patients + controls. Dot size represents –log10(adjusted p-value). Pathways with |activation z-score| ≥ 2 are color-coded to indicate predicted activation state; significant pathways with |z| < 2 are shown without activation coloring. The inflammatory and myeloid modules highlighted in Fig. 5A are preserved in the patient-only comparison. Additional stress- and nucleic-acid–sensing pathways appear in the ZNFX1 vs. interferonopathy contrast.

**Figure S5** Upstream cytokine regulator analysis across three RNA-seq contrasts

A Dot plot showing the top 20 most significant cytokine upstream regulators (adjusted p < 0.05) identified by IPA across three contrasts: ZNFX1 vs. healthy controls, ZNFX1 vs. interferonopathy patients, and ZNFX1 vs. patients + controls. Dot size represents –log10(adjusted p-value), and dot color reflects the activation z-score (only regulators with |z| ≥ 2 are color-coded). The ZNFX1 vs. interferonopathy and ZNFX1 vs. patient + control contrasts show highly concordant activation patterns for IFN-γ, TNF, and IL-1β, indicating preservation of the dominant cytokine-activation profile irrespective of whether healthy controls are included. Regulators associated with type I interferons (IFNA2, IFNB1, IFNA1/13, IFNL1) were predominantly enriched in the ZNFX1 vs. control comparison, consistent with a disease-versus-healthy baseline effect.

**References**

1.	Kawaguchi S, Higasa K, Shimizu M, Yamada R, Matsuda F. HLA-HD: An accurate HLA typing algorithm for next-generation sequencing data. Hum Mutat. 2017;38(7):788-97.
